# New Phosphorylation Sites of Rad51 by c-Met Modulates Presynaptic Filament Stability

**DOI:** 10.3390/cancers11030413

**Published:** 2019-03-23

**Authors:** Thomas Chabot, Alain Defontaine, Damien Marquis, Axelle Renodon-Corniere, Emmanuelle Courtois, Fabrice Fleury, Yvonnick Cheraud

**Affiliations:** 1 Group of Mechanism and Regulation of DNA Repair, UFIP UMR CNRS 6286/University of Nantes, 44322 Nantes, France; thomas.chabot1@univ-nantes.fr (T.C.); damien.marquis@univ-nantes.fr (D.M.); emmanuelle.courtois@univ-nantes.fr (E.C.); yvonnick.cheraud@univ-nantes.fr (Y.C.); 2 Group of Molecular Engineering and Glycobiology, UFIP UMR CNRS 6286/University of Nantes, 44322 Nantes, France; alain.defontaine@univ-nantes.fr; 3 CRCINA, INSERM, CNRS, University of Angers, University of Nantes, 44007 Nantes, France; axelle.renodon-corniere@univ-nantes.fr

**Keywords:** RAD51 recombinase, HGFR kinase, post-translational modification, DNA repair, cancer outcomes

## Abstract

Genomic instability through deregulation of DNA repair pathways can initiate cancer and subsequently result in resistance to chemo and radiotherapy. Understanding these biological mechanisms is therefore essential to overcome cancer. RAD51 is the central protein of the Homologous Recombination (HR) DNA repair pathway, which leads to faithful DNA repair of DSBs. The recombinase activity of RAD51 requires nucleofilament formation and is regulated by post-translational modifications such as phosphorylation. In the last decade, studies have suggested the existence of a relationship between receptor tyrosine kinases (RTK) and Homologous Recombination DNA repair. Among these RTK the c-MET receptor is often overexpressed or constitutively activated in many cancer types and its inhibition induces the decrease of HR. In this study, we show for the first time that c-MET is able to phosphorylate the RAD51 protein. We demonstrate in vitro that c-MET phosphorylates four tyrosine residues localized mainly in the subunit-subunit interface of RAD51. Whereas these post-translational modifications do not affect the presynaptic filament formation, they strengthen its stability against the inhibitor effect of the BRC peptide obtained from BRCA2. Taken together, these results confirm the role of these modifications in the regulation of the BRCA2-RAD51 interaction and underline the importance of c-MET in DNA damage response.

## 1. Introduction

The study of the mechanisms of DNA repair is a major challenge in the fight against cancer. Indeed, these mechanisms are often exacerbated in cancer cells. Thus, they provide radio- and chemotherapy resistance properties. Understanding DNA repair pathways allows us to consider new methods to limit this phenomenon in order to reduce tumor progression.

Homologous recombination (HR) is one of the mechanisms of DNA repair. Very well preserved during the evolution, it allows faithful repair. It is a precise and highly complex mechanism that is finely regulated. It involves many proteins whose central actor is the RAD51 recombinase. RAD51 is distributed between the cytoplasm and the nucleus but its nuclear translocation is increased in response to DNA damage with the help of the BRCA2 protein [1,2]. Following a double strand break (DSB), the resection of the 5’ end forms free 3’ end on which RAD51 is placed. Search and hybridization of the single-stranded end with the homologous sequence is ensured by the formation around the DNA of a RAD51-ATP filament essential for faithful repair [3,4]. This helical nucleofilament consists of a set of RAD51 monomers polymerized around the DNA. HR is ensured by the double association: monomer-monomer and protein-DNA.

The activity of RAD51 is precisely regulated by post-translational modifications (PTM) as well as by its association with other protein partners such as BRCA2 [5]. Among these PTMs, phosphorylation plays a major role in the regulation of the recombinase activity and may be involved in cell cycle regulation, chemoresistance and DNA damage response (DDR). Different phosphorylation sites on human RAD51 have already been identified and can involve the serine, threonine or tyrosine residues.

Indeed, a sequential phosphorylation of RAD51 on S14 by CK2 and then on T13 by Plk1 has been proven demonstrated [6]. This double phosphorylation appears to be essential for the interaction with NSB1 partner and for the recruitment of RAD51 to DNA damage sites.

Similarly, RAD51 phosphorylation on T309 by Chk1 seems to be required for the formation of RAD51 nuclear foci on DNA damage site. Interestingly, the absence of this phosphorylation increases cell sensitivity to DNA damage suggesting the importance of these PTM in the DDR biological process [7].

Actually, only one tyrosine kinase family has been described to phosphorylate RAD51 and it concerns c-ABL or its oncogenic fusion protein BCR/ABL which are able to phosphorylate RAD51 on Y315 and Y54 residues. Thus, the phosphorylation of RAD51 by c-ABL modulates its recruitment on DSB sites and its strand exchange activity [8]. In cellular context, phosphorylation of tyrosine 315 seems to promote the formation of RAD51 nucleofilament foci at the DSB site and also its nuclear translocation within the cell in response to DNA damage [9]. By using phosphomimetic approach, Subramanyam et al., have demonstrated in vitro that Y54 phosphorylation promotes RAD51 activity while Y315 phosphorylation seems to have little effect on RAD51 activities [10]. From our previous work, we have provided evidence that the phosphorylation of RAD51 by c-ABL was sequential on Y315 then Y54 which may play a pivotal role in the regulation of HR by modulating the first step of recombinase activity [8,11].

Several studies suggest that the HR pathway is modulated by receptor tyrosine kinases (RTK). For example, the inhibition of EGFR, overexpressed in many cancers, leads to a decrease in homologous recombinant repair by acting on the Ras/Raf or IRS1 [12]. Another member of the RTK family is described to affect HR, the c-MET membrane receptor (HGFR) [13,14].

HGFR is expressed in a large number of cell types (epithelial, endothelial, neural...) and plays a role mainly in embryonic development but also in carcinogenesis. It is activated by its HGF ligand, which is mainly expressed in the placenta. Ligand binding allows dimerization of the receptor and leads to its autophosphorylation on intracellular tyrosines of the receptor. This autophosphorylation triggers the tyrosine kinase activity of the receptor. Then, many signaling channels are activated in the cell. They affect mostly cell proliferation and survival.

The overexpression or the presence of constitutive mutations of the c-MET receptor, but also the overexpression of the ligand in many human cancers such as carcinomas, sarcomas and glioblastomas is related to significant morbidity [15,16,17,18]. In addition, c-MET and EGFR are able to heterodimerize to induce cross-activation of the two receptors by one of the two ligands, resulting in an even greater number of activated signaling pathways [19]. Finally, endocytosis of the c-MET tyrosine kinase receptor is also observed following its activation that would explain the phosphorylation of cytosolic proteins [20]. With regard to the homologous recombination repair mechanism, it has been shown that c-MET inhibition decreases the formation of the RAD51/BRCA2 complex [13] to lead to a reduction in HR DSB repair. Other experiments have also shown that the use of c-MET phosphorylation inhibitors reduces the half-life of proteins that play a role in DNA repair, such as RAD51 [21].

Our study shows, for the first time, the existence of a direct link between RAD51, the key protein for DNA repair by HR, and a membrane receptor with tyrosine kinase activity, c-MET. By combining in vitro and in silico approaches, we show that c-MET is able to phosphorylate RAD51 on several tyrosine residues. These phosphorylations are closely related to the oligomeric state of RAD51 and to its interaction with the BRC motif of BRCA2. Our results suggest that c-MET-mediated RAD51 phosphorylation could play a potential role in the BRCA2-dependent regulation of RAD51.

## 2. Results

### 2.1. c-MET Linase Is Able to Phosphorylate RAD51 in Vitro

**Figure 1:**
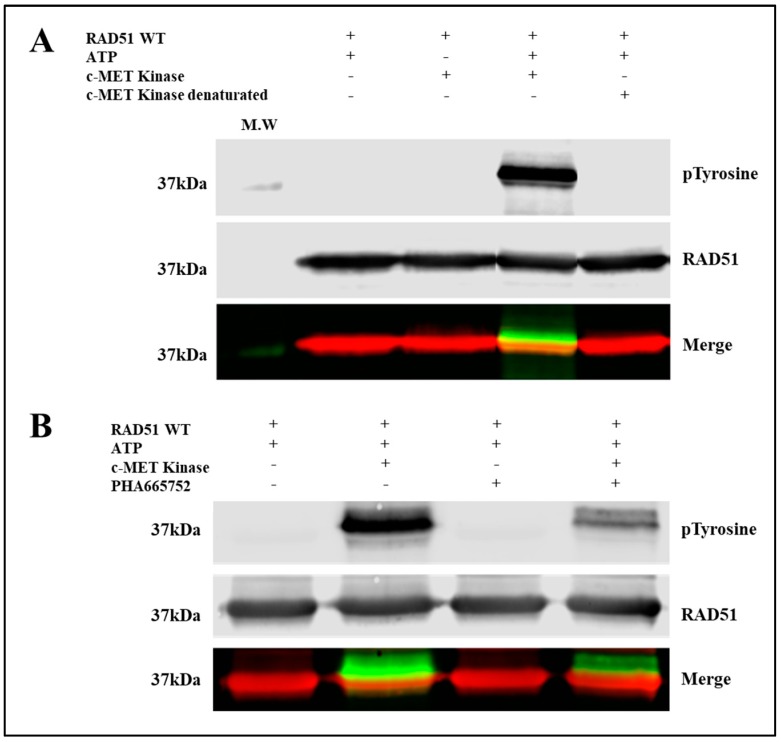
c-MET phosphorylates RAD51 in vitro. (**A**) Purified RAD51 WT protein (10 µM) was incubated with ATP (2 mM) and with or without the active C-terminal part of the c-MET kinase (0.1 µM). Then the proteins were separated by SDS-PAGE 12% gel and analyzed by western blot using antibodies (red: anti-RAD51, green: anti-phosphotyrosine) (M.W: molecular weight). (**B**) The same experiment was performed with or without a specific c-MET kinase inhibitor, PHA665752 (1 µM). Each of the experiments was conducted three times independently (*n* = 3) and the blots presented above correspond to a representative experiment.

To evaluate the phosphorylation of RAD51 in vitro, we used purified RAD51-WT recombinant proteins and the C-terminal catalytic domain of c-MET. This catalytic domain is indeed constitutively active even in the absence of the HGF ligand because tyrosines of the catalytic domain are phosphorylated [22]. Interestingly, HGF ligand binding promotes dimerization and cellular internalization of the receptor which is then degraded in the cell [23]. Moreover the presence of the C-terminal domain of c-MET receptor into the nucleus has been shown [22] that supports our biochemical approach. In the Figure 1, the anti-phosphotyrosine does not recognize RAD51 in absence of c-MET or in presence of denatured c-MET, confirming the absence of phosphorylated RAD51 form. In addition, purified RAD51 proteins were expressed in bacterial system (*E. coli*) that ensures the production of non-phosphorylated RAD51.

Immunoblot analysis showed a significant increase in the phosphorylation state of a protein at 37 kDa, revealed by both the anti-phosphotyrosine antibody and the antibody directed against the RAD51 protein (Figure 1A). This superposition of signals suggests that the c-MET kinase phosphorylates the RAD51 protein in the presence of ATP. The absence of kinase or the use of inactivated kinase by thermal denaturation or the absence of ATP leads to the disappearance of the phosphorylated form of RAD51 confirming the direct phosphorylation of the RAD51 protein by the c-MET kinase in vitro.

A specific c-MET kinase inhibitor, PHA665752, was also used to test the specificity of this phosphorylation. PHA665752 is a competitive ATP inhibitor that fits into the ATP pocket of the c-MET receptor at residues I1084-K1110 [13]. A significant decrease of approximately 60% in the phosphorylation level of the RAD51 protein was observed when 1 µM inhibitor was used (Figure 1B). These results confirm that phosphorylation of RAD51 is directly related to c-MET activity. RAD51 is therefore a substrate that is recognized and phosphorylated by the c-MET kinase in vitro.

### 2.2. c-MET Phosphorylates Four Tyrosines on RAD51

Figure 1 shows the presence of several phosphorylated RAD51 delayed bands suggesting the existence of several phosphorylations of RAD51. Therefore, we found it is interesting to identify the number and positions of tyrosines phosphorylated by c-MET.

We first used several phosphorylation prediction softwares (see Materials and Methods section). The results suggest that among all RAD51 tyrosines, six are potentially phosphorylatable by c-MET: Y159, Y178, Y191, Y205, Y301 and Y315.

To verify and validate these bioinformatic prediction results, two experimental approaches were used, the first by site-directed mutagenesis of tyrosine residues of RAD51 and the second by mass spectrometry.

Using site-directed mutagenesis, each tyrosine residue of interest was replaced by a phenylalanine residue. This leads to the production and purification of non-phosphorylatable RAD51 mutants with a purity of over 95% (Figure S1). Indeed, the replacement of a tyrosine residue with a phenylalanine residue makes the addition of a phosphate group to this residue impossible. In addition, the structure of these two aromatic residues is extremely close, consequently the secondary structure of the mutant proteins remains identical to the wild form. Moreover, we verified the conservation of the secondary structure of all the mutants by circular dichroism and we confirmed the absence of structural changes of the mutated forms compared to the wild form (Figure S2).

**Figure 2:**
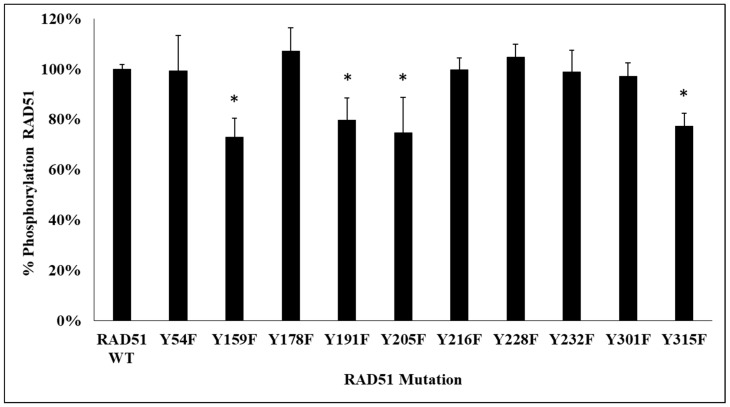
Decrease in RAD51 phosphorylation level in 4 RAD51 mutants. RAD51 WT protein and all non-phosphorylatable mutants of RAD51 (10 µM) were purified and incubated in the presence of kinase-active c-MET (0.1 µM) and ATP (2 mM). Then, the proteins were separated by SDS-PAGE 12% gel and analyzed by western blot using anti-RAD51 and anti-phosphotyrosine antibodies. The signals were quantified with the Odyssey scanner and compared with the signal from RAD51 WT. The graph represents the phosphorylation percentage of each mutant compared to the wild protein and obtained from the quantification of blots. Each experiment was conducted three times independently (*n* = 3; error bars: s.d.; * *p* < 0.05).

Each of the mutant and wild type proteins tagged with a Histidine tag was produced in *E. coli* BL21-DE3 bacteria and then purified on a NiNTA column. After the production of 11 proteins (mutants and wild type), in vitro phosphorylation assays using the c-MET kinase were performed (Figure 2).

The phosphorylation signals, revealed by the anti-phosphotyrosine antibody, were qualified. We observed a significant decrease of phosphorylation of about 25% for 4 non-phosphorylatable Y/F mutants, compared to the wild-type RAD51 protein. The mutated tyrosines were Y159, Y205, Y191 and Y315.

The second approach was to confirm the phosphorylation of these sites on the RAD51 protein by mass spectrometry (MS). A migration of RAD51 phosphorylated by c-MET was carried out on SDS-PAGE gel. Staining with Coomassie blue G-250 allowed the identification of the bands which were then stored in a H2O/Acetic acid 10% medium. MS analysis confirmed the phosphorylation of 3 of the 4 previously obtained sites: Y159, Y191 and Y205.

By combining all the results obtained by these different methods of biostatistics, biochemistry and biophysics, we concluded that the in vitro phosphorylation of RAD51 by the c-MET kinase occurs at four tyrosines: Y159, Y191, Y205 and Y315.

### 2.3. C-MET Phosphorylation is Dependent on the Structural Organization of RAD51

**Figure 3:**
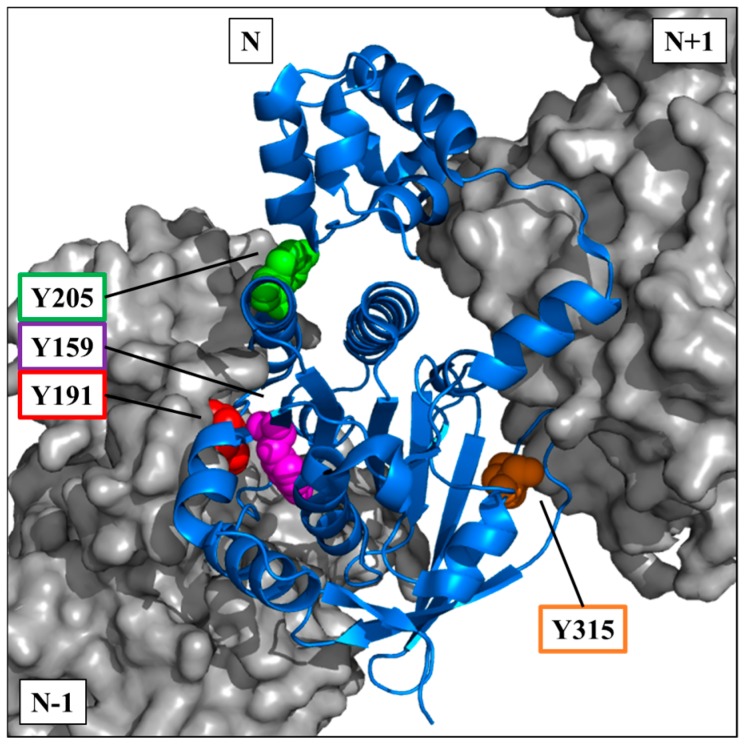
Position of potential phospho-tyrosine residues in the RAD51 filament. Using the PyMOL software and with the nucleofilament structure of RAD51 [24], tyrosines Y159 (magenta), Y191 (red), Y205 (green) and Y315 (orange) of a monomer N of RAD51 (blue—cartoon) are indicated. The monomers N+1 and N-1 are also represented (grey—surface).

The crystallographic data of the RAD51 nucleofilament obtained by Brouwer et al. [24], led us to localize the four tyrosine residues potentially phosphorylatable by c-MET. They are all present in the C-terminal domain of the RAD51 protein (Figure 3).

The visualization on a trimer of RAD51 allowed us to position the four residues in the central monomer (indicated as N in the Figure 3). Three of four sites are in the vicinity of the monomer-monomer interface of RAD51 in the nucleofilament. Residues Y159, Y191 and Y205 appear to be at the interface of the N-1/N monomers, while Y315 appears to be at the interface of the N/N+1 monomers (Figure 3).

The position of these residues suggests that their exposure and therefore accessibility depend on the structural organization of RAD51. Therefore, tyrosine residues would be more exposed in the monomeric forms than in the oligomeric forms of RAD51.

To test and confirm this hypothesis, we evaluated the effect of the oligomeric state of RAD51 on its phosphorylation by in vitro phosphorylation experiments using the RAD51 F86E and A190/192L mutants, which are described as no-self associated mutants [25,26].

**Figure 4:**
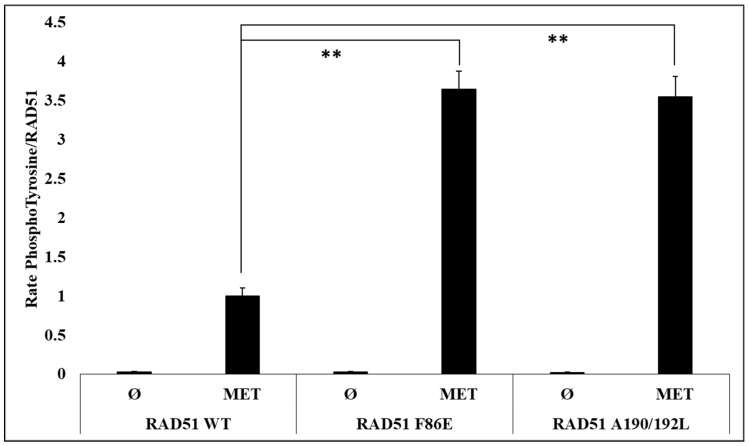
Phosphorylation level is promoted for monomeric RAD51 mutant variants. The RAD51 WT protein (10 µM) and all non-polymerizable mutants of RAD51: F86E and A190/192L [26] were purified and incubated in the presence of kinase-active c-MET (0.1 µM) and ATP (2 mM). Then, the proteins were separated by SDS-PAGE 12% gel and analyzed by western blot using anti-RAD51 and anti-phosphotyrosine antibodies. The signals were quantified with the Odyssey scanner and compared with the signal from RAD51 WT. The graph represents the phosphorylation rate of each mutant compared to the wild type protein following the quantification of blots from three independent experiments (ø: without kinase) (*n* = 3; error bars: s.d.; ** *p* < 0.01).

After quantification of the RAD51 phosphorylation rate signals by western blot, we observed a significant (3.5 fold) increase in the phosphorylation of the two mutants compared to the RAD51-WT protein (Figure 4).

**Figure 5:**
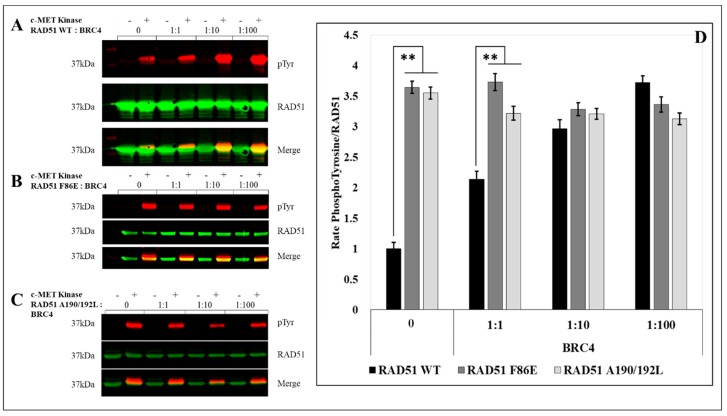
c-MET-mediated RAD51 phosphorylation is related to its oligomeric state. (**A**) The RAD51 WT protein (10 µM) and all non-polymerizable mutants of RAD51: (**B**) F86E and (**C**) A190/192L were purified and incubated with ATP (2 mM), with or without kinase-active c-MET (0.1 µM), and an increasing concentration of BRC4 peptide. Then the proteins were separated by SDS-PAGE 12% gel and analyzed by western blot using anti-RAD51 (green) and anti-phosphotyrosine (red) antibodies. The signals were quantified by Odyssey scanner and compared with the signal from RAD51 WT. (**D**) The graph represents the phosphorylation rate of each mutant compared to the wild protein following the quantification of blots from three independent experiments (*n* = 3; errors bars: s.d.; ** *p* < 0.01).

The RAD51 protein is normally present in monomer-oligomer equilibrium [25]. We used the BRC4-28 peptide which is an inhibitor of the polymerization of RAD51. It is able to shift the equilibrium in favor of the monomeric form of RAD51 [27,28,29,30]. We then performed supplementary in vitro phosphorylation tests with RAD51-WT protein in presence of BRC4-28. The PhosphoRAD51/RAD51 ratios were calculated from the anti-phosphotyrosine and anti-RAD51 immunoblots (Figure 5A). We observed that the incubation with increasing concentrations of BRC4-28 peptide increased the proportion of phosphorylation of RAD51 to reach the maximum value of 4 (Figure 5D). This result suggests that peptide-induced depolymerization of RAD51 promotes its phosphorylation by c-MET.

We also performed the same type of experiment with the no-polymerizable mutants F86E (Figure 5B) and A190/192L (Figure 5C). While the BRC4-28 peptide promotes the phosphorylation of RAD51-WT, no significant variation was observed with RAD51 F86E and A190/192L non-polymerizable mutants. It is interesting to note that the increase in RAD51-WT phosphorylation tends to similar values to those observed for the two non-polymerizable mutants (Figure 5D).

This convergence of phospho/non-phospho ratios demonstrates that the phosphorylation of RAD51 by c-MET is increased with the monomeric forms and emphasizes the importance of the polymerization state on the phosphorylation level of RAD51 by c-MET.

### 2.4. RAD51 Phosphorylation by c-MET Promotes its Multimeric State

**Figure 6:**
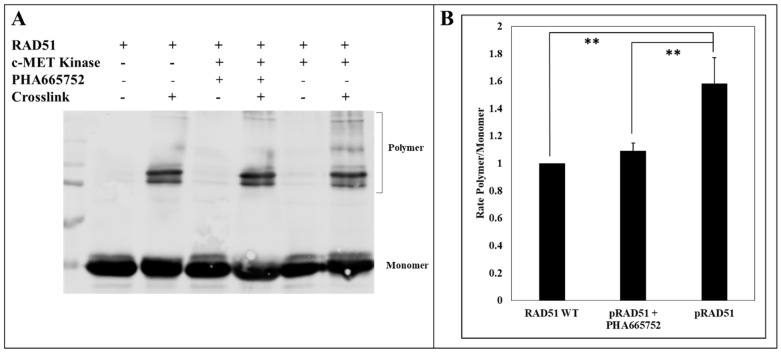
c-MET-mediated RAD51 phosphorylation promotes the formation of high-molecular-weight RAD51 oligomers. (**A**) The RAD51 WT protein (10 µM) was purified and incubated with ATP (2 mM) and with or without kinase-active c-MET (0.1 µM) and PHA665752 inhibitor (1 µM). Then, the proteins were crosslinked with BS3, separated by SDS-PAGE 10% gel and analyzed by western blot using anti-RAD51 antibodies. The signals were quantified by Odyssey scanner. (**B**) The graph represents the average of the ratios of the polymer forms of RAD51 phosphorylated or not by c-MET. The ratios were obtained after quantification of the blots from three independent experiments (*n* = 3; error bars: s.d.; ** *p* < 0.01).

There is a tight relationship between the oligomerization and phosphorylation state of RAD51. To find out whether the level of oligomerization of RAD51 changes when the protein is phosphorylated, we carried out a crosslinking assay of the oligomer forms of RAD51 before and after phosphorylation. The proteins were then analyzed by SDS-PAGE and immunoblotting. Revelation with an anti-RAD51 antibody shows that RAD51 phosphorylation by c-MET leads to an increase in multimer forms of RAD51 (Figure 6A). Indeed, after quantification, the proportion of multimer forms of RAD51 is 1.6 times higher after phosphorylation (Figure 6B). Moreover as shown in Figure 6, the inhibition of kinase activity of c-MET by PHA665752 inhibitor restores the polymerization status of RAD51 in comparison of RAD51 with active c-MET. This experiment confirms that the c-MET-mediated phosphorylation of RAD51 promotes the oligomer form. These results suggest that tyrosine phosphorylation of RAD51 by c-MET leads to an increase in the polymerization state of the recombinase.

### 2.5. RAD51 Phosphorylation by c-MET Does not Influence the Formation of the Presynaptic Filament but Enhances its Stability Against the BRC4 Peptide

Is the structural equilibrium of RAD51 closely related to its phosphorylation, what could be the effect of phosphorylation on the RAD51 nucleofilament? Nucleofilament formation, which is highly dependent on the self-association capacity of RAD51, is indeed an essential step in HR.

**Figure 7:**
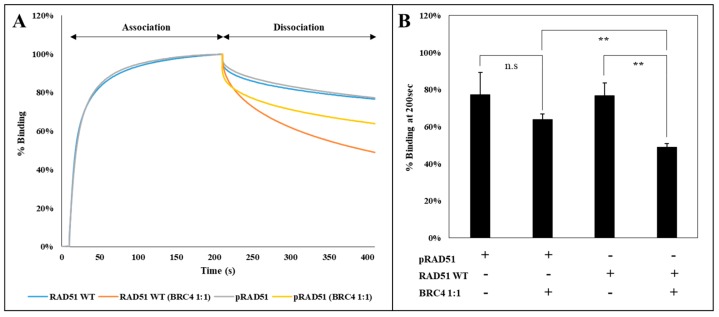
BRC4-mediated nucleofilament dissociation is less effective when RAD51 is phosphorylated. The RAD51 WT protein (10 µM) was purified and phosphorylated in vitro by kinase-active c-MET (0.1 µM). Then, we incubated a streptavidin associated-tip with polydT nucleic acids to the extracts diluted in a phosphate-ATP buffer. The protein-DNA interaction was measured by interferometry (Blitz®). After 10 s of baseline, the different RAD51 protein samples were added and the association and dissociation phase were then recorded for 200 s. Dissociation was performed with a conventional phosphate buffer with or without BRC4 peptide (1:1). (**A**) The experiment was repeated four times to obtain the curve representing the average of the performed experiments (*n* = 4). (**B**) The impact of BRC4 on the dissociation of RAD51 to DNA was achieved at t = 200s of dissociation. The graph shows the percentage of association of the different RAD51 proteins with nucleic acids after the addition or not of BRC4. The average values of the each experiments were compared to the wild protein (*n* = 4; error bars: s.d.; ** *p* < 0.01; n.s: *p* > 0.05).

To analyze the effect of RAD51 phosphorylation by the c-MET kinase on this step, we used an interferometry interaction test (BLItz^®^). A biotinylated single-stranded DNA was immobilized on a streptavidin tip, and then incubated in a solution containing phosphorylated or non-phosphorylated RAD51 protein. This approach enabled us not only to evaluate the association of the phosphorylated RAD51 protein with the DNA but also to follow the dissociation of the DNA with or without the BRC4 peptide. Concerning the association phase of RAD51 with DNA, we observed that there is no difference between the phosphorylated and non-phosphorylated form (Figure 7A). This is also the case when dissociating phosphorylated and non-phosphorylated RAD51 from DNA using a dissociation buffer. This result is confirmed by measuring the dissociation constants KD of each protein regarding its interaction with DNA. We estimated similar KD values: KD RAD51 = 1.28 × 10^−7^ M and KD pRAD51 = 1.41 × 10^−7^ M.

By contrast, Figure 7 shows that there is a difference in the dissociation of the RAD51 nucleofilament induced by the BRC4-28 peptide depending on whether RAD51 is phosphorylated or not. Quantification of phosphorylated and non-phosphorylated RAD51 binding on the DNA in percentage after 200 sec shows that the dissociation is two times lower when RAD51 is phosphorylated (Figure 7B). This is confirmed by the determination of the dissociation rate constants. It is lower for the phosphorylated form koff pRAD51 = 3.32 × 10^−7^ M than for the unphosphorylated form koff RAD51 = 4.56 × 10^−7^ M.

The nucleofilament or presynaptic filament of RAD51 is capable of locating a homologous duplex DNA molecule and catalyzing the invasion of the duplex to form a DNA displacement loop called the “D-loop” [31]. We have estimated the effect of phosphorylation on the performance of RAD51 by performing a D-loop test which allows the estimation of the invasion and homologous pairing step of RAD51 activity.

**Figure 8:**
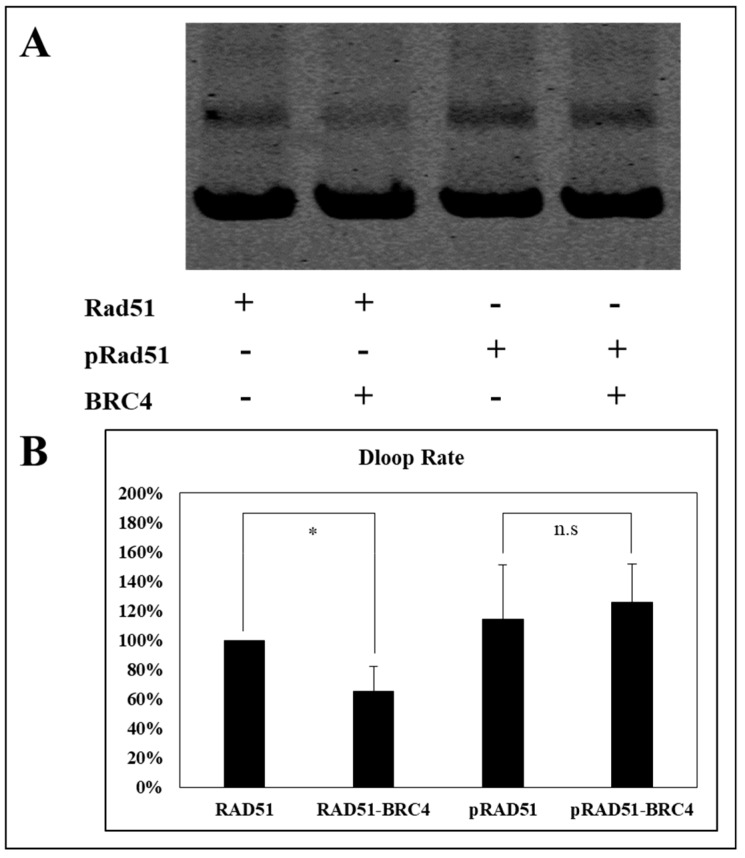
Invasion and homologous pairing steps are less sensitive to the BRC4 peptide when RAD51 is phosphorylated. (**A**) The RAD51 WT protein (10 µM) was purified and phosphorylated in vitro by kinase-active c-MET (0.1 µM). The protein was incubated with the D-loop mix containing fluorophore labelled ssDNA to allow nucleofilament formation. The BRC4 peptide was then added (1:1) before the addition of the unlabelled plasmid allowing the formation of a D-loop. The whole was separated on 1% agarose gel. The signals were quantified by Odyssey scanner. (**B**) The graph represents the percentage of formed D-loop complexes following the phosphorylation or not of RAD51 by c-MET. Blots from three independent experiments were used for quantification (*n* = 3; error bars: s.d.; * *p* < 0.05; n.s: *p* > 0.05).

As shown in Figure 8, the BRC4-28 peptide decreased the percentage of D-loop activity (about 40%) with the non-phosphorylated RAD51 form. This inhibitory effect is significantly abrogated when RAD51 was phosphorylated by c-MET. Indeed, we observed no change of D-loop activity of phosphorylated RAD51 in the presence and in absence of the BRC4-28 peptide (Figure 8). These results suggest that the presynaptic filament involving phosphorylated RAD51 is more stable and less sensitive to the BRC4-28 peptide than the non-phosphorylated RAD51 form.

### 2.6. Activation of c-MET by HGF Increases the Repair of Double-Strand Breaks by Homologous Recombination

**Figure 9:**
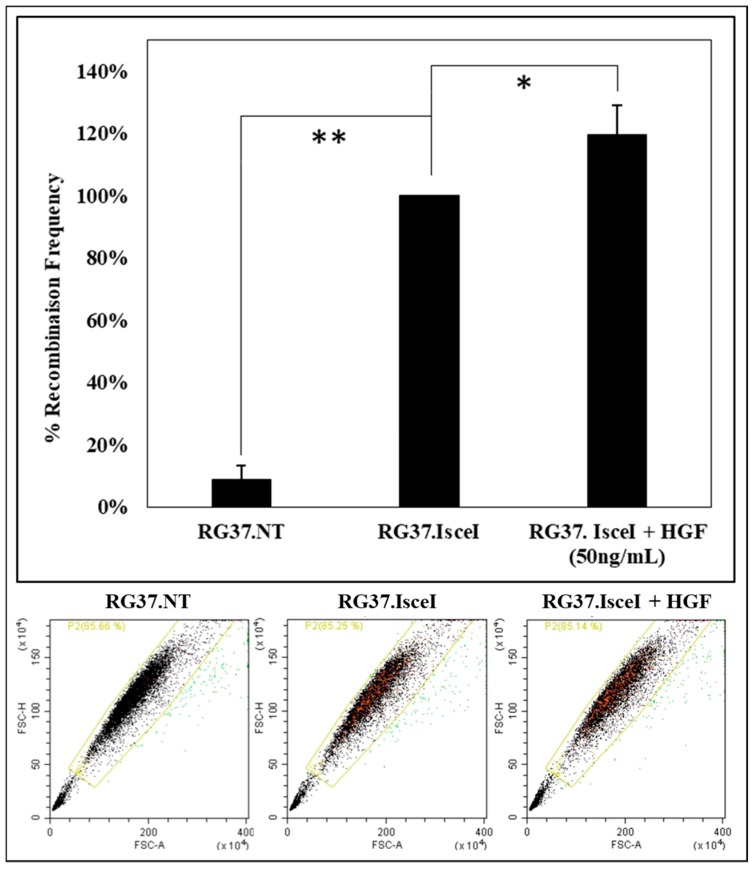
Activation of c-MET by HGF increases the repair of double-strand breaks by homologous recombination. Frequency of HR in DR-GFP RG37 cells pre-treated or not with HGF (50ng/mL for 24h) is estimated by DR-GFP assay. The data represent the mean of the percentage recombination frequency of three independent experiments (*n* = 3; error bars: s.d; * *p* < 0.05; ** *p* < 0.01). Representative diagrams of RG37 expressing reconstituted GFP protein (population in green) and cells negative for GFP (main population).

An analysis of the effect of c-MET receptor activation by HGF on HR was performed. We used a cell model RG37 I-SceI that allows us to quantify HR by measuring the fluorescence of the “repaired” GFP protein. Cells are analyzed with a cytometer, GFP fluorescence is more important when the I-SceI endonuclease is expressed (Figure 9).

If the cells are pretreated for 24 h with HGF (50 ng/mL), a significant increase of about 20% of recombination frequency is observed (Figure 9). Similar work has also shown a decrease in HR when c-MET is inhibited [13]. Therefore, this result provides evidence of the relation between this RTK and HR, suggesting a key role of RAD51.

## 3. Discussion

In the last decade, DNA repair pathways have been described as promising targets in cancer therapy [31]. Understanding the regulation of DNA repair proteins is essential to overcome anticancer resistance. The phosphorylation of the RAD51 recombinase is a well-established factor that could modulate HR in DNA repair. Among the kinases capable of phosphorylating RAD51, the receptor tyrosine kinase family appears to act on the DNA repair pathways including HR. Interestingly, overexpression and constitutive activation of RTK are common in a number of cancer types and are suspected to have serious effects on the DNA repair pathways.

Previous results highlight the possible connection between c-MET receptor and RAD51 since kinase inhibition reduces the protein level of RAD51 [19] and induces a decrease of HR repair in response to DNA damage [13]. Activation of the c-MET receptor involves its ability to self-phosphorylate and leads to the phosphorylation of many protein partners that play a role in a large number of cell signaling pathways such as cell proliferation and survival via the MAPK, GAB1, STAT3 and PI3K/AKT pathways [32,33,34]. Our results demonstrate that c-MET kinase can also phosphorylate RAD51 at four tyrosines: Y159, Y191, Y205 and Y315 (Figure 1 and Figure 2).

The activity of RAD51 involves many protein-protein interactions which determine the function and the role of RAD51 in the DNA repair context. The RAD51 protein presents different oligomeric forms in buffer solution [35,36]. Structure resolutions of RAD51 in different organisms show that it is frequently organized in a ring with 6 to 8 monomers [37,38], and even in short filaments depending on the buffer composition [39]. The equilibrium between these different forms is also dependent on the protein concentration [35] and the presence of other molecules like peptides, salt or co-factors.

Nucleotide cofactors are also important elements to consider as they modulate the equilibrium and configuration of RAD51 oligomers. Schay et al. [39] demonstrate the role of ATP in the specific conformation of RAD51. By electron microscopy, they show that the binding of ATP modifies the protein-protein interfaces and leads mainly to oligomeric structures in the form of rings or short helices. These filament and ring structures were also observed by Baumann [40] in the absence of ATP. The ring structure was predominant when the salt concentration was increased. All our experiments were carried out in saturating concentrations of ATP (2 mM) in order to avoid the formation of disordered filaments as described by Shay et al. [39]. Moreover, the biologically active oligomer form requires ATP [41].

The self-association of RAD51 involves a polymerization motif located in its N-terminal domain. Mutations in this region such as F86E, A89E lead exclusively to monomeric forms and thus to the abrogation of the self-association of RAD51 [25,26].

By using non-polymerizable mutants, we have shown that the level of RAD51 phosphorylation by c-MET is strongly related to the oligomeric state of RAD51 (Figure 4 and Figure 6).

This RAD51 monomer-monomer self-association property holds a prominent place in the formation of the RAD51 filament on single-stranded DNA (ssDNA). Disturbance in the monomer-monomer interface may impact nucleofilament formation and thus recombinase activity.

By combining in silico prediction, mass spectroscopy (MS) and biochemical approaches, we identified four phosphorylatable tyrosines (Y159, Y191, Y205, and Y315) by c-MET kinase.

It is interesting to note that the Y315 phosphorylation detection failed by MS method. In our previous work, we proposed an explanation of this absence of detection. After proteolysis of RAD51 by trypsin, the length (28 amino acids) of Tyr315-containing peptide and its negative charge (pHi = 4.03) could interfere with its detection by MS [8]. Besides, in another work [42], Y315 phosphorylation mediated by c-ABL kinase was not detected by MS while the Y54 phosphorylation was done, highlighting the difficulty to analyze this singular peptide.

The four phosphorylatable tyrosine residues that we identified in this study are all located in these monomer-monomer interfaces N-1/N or N/N+1 or in their vicinity (Figure 3). The Y191 and Y315 tyrosines have, moreover, been described in the literature as hinge positions at the monomer-monomer interfaces of RAD51 [43,44]. Using molecular modelling approaches, the addition of a phosphate residue can be carried out through ionic bond with the lysine or the arginine of monomer N + 1 as described in Alligand et al. [11]. The generation of new bonds between RAD51 monomers could explain why phosphorylation increases the oligomer state of RAD51.

Although our findings show that the phosphorylation of tyrosine residues seems to strengthen monomer/monomer interactions by promoting the oligomeric state of RAD51, the nucleofilament formation does not seem to be affected by these phosphorylations. Indeed phosphorylated RAD51 binds to single stranded DNA and exhibits a dissociation constant (KD = 140 nM) similar to the binding affinity of non-phosphorylated RAD51 (KD = 128 nM). Theses KD values are in the same order of magnitude and are consistent with the data in the literature [45].

The BRC4-28 peptide inhibitor is able to destabilize the multimeric forms of RAD51. It is derived from the BRCA2 protein and it is capable of mimicking monomer-monomer interfaces and causes also the dissociation of the nucleofilament of RAD51 [38]. Our results have shown that phosphorylation abrogates the inhibitor effect of BRC4-28 on the nucleofilament stability (Figure 5 and Figure 7).

It is noteworthy that this peptide corresponds to the BRC interaction motif of the BRCA2 protein, which has a central role during HR through controlling the RAD51 recombinase. BRCA2 contains eight BRC motifs (from BRC1 to BRC8) which are able to bind directly to RAD51 with varying affinity [46]. Therefore, BRCA2-RAD51 interactions have an essential role on biological events including the intracellular distribution of RAD51 in undamaged and damaged cells, the recruitment of RAD51 on DNA damage sites and on RAD51 oligomerization.

**Figure 10:**
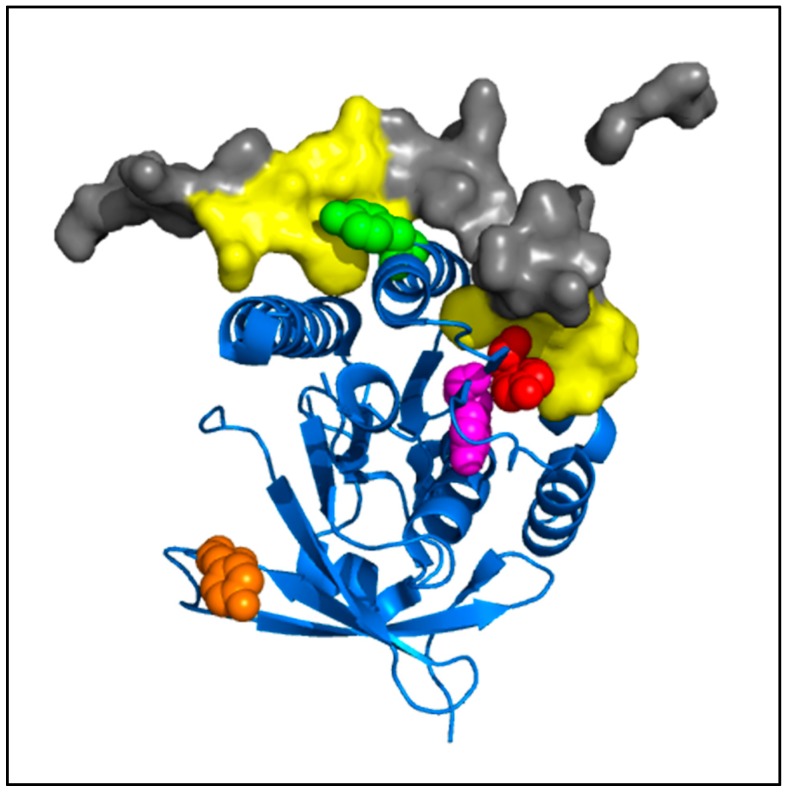
Modeling of tyrosine residues of RAD51 involved in the RAD51-BRC4 interface. Using the PyMOL software and the structure of the C-terminal part of RAD51 with BRC4 [25], tyrosines (Y159, magenta; Y191, red; Y205, green; Y315, orange) were indicated. The BRC4 peptide [26] and its high interaction areas with RAD51 are also represented (BRC4, grey, surface; high interaction with RAD51, yellow, surface).

The low sensitivity of the phosphorylated RAD51 forms to the BRC4-28 inhibitor can be explained by the localization of the RAD51 interaction site and by the formation of new bonds generated by phosphate groups on tyrosine residues. Molecular modelling of RAD51-BRC4 (Figure 10) shows that the Y159, Y191 and Y205 residues localized at the N-1/N interface (Figure 3) are directly in contact with the peptide. Two regions of the BRC4 peptide (Zone 1: F1524–K1530; Zone 2: K1541–E1548) are known to have high affinity with RAD51 protein (yellow, Figure 10). The negative charge and the steric repulsion of phosphotyrosine residues can prevent RAD51-BRC4 interaction.

This study shows that the recombinase activity of RAD51 of presynaptic filaments containing phosphorylated RAD51 was less sensitive to the inhibitory effect of the BRC4 peptide. These results highlight the importance of the BRCA2-RAD51 interaction in cells and can explain the impact of c-MET activation on homologous recombination repair where BRCA2 and RAD51 are confidentially connected.

On the other hand, it is difficult to assess the contribution and effects of each of the four phosphorylatable sites on RAD51 activity. According to the literature Y315 phosphorylation has little effect on the in vitro RAD51 activities [10] suggesting that BRCA2-mediated effects could be associated with one or more of the three sites of the N-1/N interface or the RAD51/BRC4-28 interface.

Our preliminary cellular result (Figure 9) shows that the c-MET activation by its natural ligand HGF increases the DNA repair by HR through the phosphorylation of RAD51. For the first time, we provide evidence that the direct relationship between c-MET and RAD51 could modulate the DNA repair pathway by HR and that could open new perspectives for anticancer therapeutic strategy.

## 4. Materials and Methods

### 4.1. Phosphorylation Site Prediction

The FASTA Sequence of RAD51 was uploaded on 3 web sites (GPS 3.0—gps.biocuckoo.org, Phosphonet—phosphonet.ca, Networkin—networkin.info) to predict six RAD51 phosphorylation sites induced by c-MET kinase activity after data compilation.

### 4.2. In Silico Modelisation

PyMOL 2.0 software was used for modelling of RAD51 and its mutation or phosphorylation. We used PDB Structure 5nwl [24] and 1n0w [25].

### 4.3. Production and Purification of RAD51 Proteins

The cDNA of His-tagged RAD51 was cloned in pET15b vector (Novagen-Merck, Darmstadt, Germany). All RAD51 Y/F, RAD51 F86E and RAD51 A190/192L were generated by site-directed mutagenesis using overlapping oligo-nucleotides. The DNA sequences of the mutant clones were checked and validated. RAD51-WT and RAD51 mutants were then over-expressed in *Escherichia coli* BL21- DE3 strain at 37 °C. The bacteria are then lysed in buffer (Tris HCl 50 mM, NaCl 500 mM, glycerol 10%, β-mercaptoethanol 5 mM, imidazole 5 mM). His-tagged RAD51-proteins were purified on a NiNTA resin (#P6611, Sigma, St Louis, MO, USA) (washing buffer: Tris HCl 50 mM, NaCl 500 mM, glycerol 10%, β-mercaptoethanol 5 mM, increasing imidazole concentration). Imidazole used for purification was removed by dialysis (dialysis buffer: Tris HCl 50 mM, NaCl 500 mM, glycerol 10%, EDTA 1 mM, DTT 1 mM, decreasing imidazole concentration). Protein concentrations were determined by the Bradford assay. The purity of proteins was analyzed by SDS- PAGE followed by Coomassie staining. All His-tagged RAD51 proteins were kept at −80 °C prior to use.

RAD51-Y54F-for: ggaggctgttgcctttgcgccaaagaagg

RAD51-Y54F-rev: ccttctttggcgcaaaggcaacagcctcc

RAD51-Y159F-for: gaaggaaaggccatgttcattgacactgaggg

RAD51-Y159F-rev: ccctcagtgtcaatgaacatggcctttccttc

RAD51-Y178F-for: ggcagtggctgagaggtttggtctctctggcagtg

RAD51-Y178F-rev: cactgccagagagaccaaacctctcagccactgcc

RAD51-Y191F-for: cctggataatgtagcatttgctcgagcgttcaacac

RAD51-Y191F-rev: gtgttgaacgctcgagcaaatgctacattatccagg

RAD51-Y205F-for: cagacccagctcctttttcaagcatcagccatg

RAD51-Y205F-rev: catggctgatgcttgaaaaaggagctgggtctg

RAD51-Y216F-for: gccatgatggtagaatctaggtttgcactgcttattgtagactg

RAD51-Y216F-rev: cagtctacaataagcagtgcaaacctagattctaccatcatggc

RAD51-Y228F-for: gtgccaccgcccttttcagaacagactactcg

RAD51-Y228F-rev: ccgagtagtctgttctgaaaagggcggtggcac

RAD51-Y232F-for: ccctttacagaacagacttctcgggtcgaggtgagc

RAD51-Y232F-rev: gctcacctcgacccgagaagtctgttctgtaaaggg

RAD51-Y301F-for: cccatgcatcaacaaccagattgtttctgaggaaaggaagagggg

RAD51-Y301F-rev: cccctcttcctttcctcagaaacaatctggttgttgatgcatggg

RAD51-Y315F-for: cagaatctgccaaatcttcgactctccctgtcttc

RAD51-Y315F-rev: gaagacagggagagtcgaagatttggcagattctg

RAD51-F86E-for: ctcgtgcccatgggcgagaccacggcgacggag

RAD51-F86E-rev: ctccgtcgccgtggtctcgcccatgggcacgag

RAD51-A190/192L-for: cctggataatgtactgtatctgcgagcgttcaacac

RAD51-A190/192L-rev: gtgttgaacgctcgcagatacagtacattatccagg

### 4.4. RAD51 Protein Sequence and Position of Tyrosine Residues

MAMQMQLEANADTSVEEESFGPQPISRLEQCGINANDVKKLEEAGFHTVEAVA**Y**APKKELINIKGISEAKADKILAEAAKLVPMGFTTATEFHQRRSEIIQITTGSKELDKLLQGGIETGSITEMFGEFRTGKTQICHTLAVTCQLPIDRGGGEGKAM**Y**IDTEGTFRPERLLAVAER**Y**GLSGSDVLDNVA**Y**ARAFNTDHQTQLL**Y**QASAMMVESR**Y**ALLIVDSATAL**Y**RTD**Y**SGRGELSARQMHLARFLRMLLRLADEFGVAVVITNQVVAQVDGAAMFAADPKKPIGGNIIAHASTTRL**Y**LRKGRG ETRICKI**Y**DSPCLPEAEAMFAINADGVGDAKD.

### 4.5. Circular Dichroism Measurement

The circular dichroism (CD) spectra of purified RAD51 protein were measured using a J-810 spectropolarimeter (Jasco, Mary’s Court Easton, MD, USA) and a mini-quartz cell with a path length of 0.2 cm. The spectra were recorded in the range from 210 to 260 nm and averaged over five scans to increase the signal-to-noise ratio (response time, 0.125 s; resolution, 0.1 nm). The final concentration of each RAD51 protein was 4 µM in phosphate buffer saline (PBS), pH 7.4.

### 4.6. c-MET Kinase Assay

Recombinant active c-MET (0.1 µM) (#0171-0000-1, ProQinase, Freiburg, Germany) was incubated with 2 mM of ATP and 10 µM of recombinant RAD51 (WT or mutant) in 25 µL kinase buffer (60 mM HEPES-NaOH, pH 7.5; 3 mM MgCl_2_; 3 mM MnCl_2_; 3 μM Na-orthovanadate; 1.2 mM DTT; 50 μg/mL PEG20.000). Reactions were incubated at 30 °C for 1 h and terminated by addition of Laemmli SDS sample dilution buffer. Proteins were separated by SDS-PAGE 12% gel, and phosphorylation was visualized by immunoblot. The c-MET inhibitor, PHA665752 (#S1070, Selleckchem, Houston, TX, USA) was used during the c-MET kinase Assay at 1 µM.

### 4.7. Chemical Cross-Linking and Polymerization Assay

RAD51 protein (10 μM) was cross-linked by incubation with 20 mM BS3 (#21580, ThermoFisher, Waltham, MA, USA) at room temperature for 30 min. Cross-linking was terminated by adding Tris 1M pH 8.0 for 15 min and analyzed by SDS-PAGE 10% gel and western blot.

### 4.8. Immunoblot Analyses

Samples were separated by electrophoresis on 12% SDS–polyacrylamide gels and transferred to nitrocellulose membranes (GEHealthcare, Little Chalfont, UK). Membranes were blocked for 1 h in 0.1% TBS-Tween20 containing 5% BSA. Primary antibodies were rabbit anti-RAD51 (ABE257, Merck Millipore, Darmstadt, Germany & Prestige, Sigma, St Louis, MO, USA) or mouse anti-pTyr (#9411S, Cell Signaling, Danvers, MA, USA). Membranes were washed three times with 0.1% TBS-Tween20 for 10 min and incubated for 1 h with anti-mouse Alexa-Fluor680-conjugated or anti-rabbit Alexa-Fluor800-conjugated secondary antibodies (#A-32735, Invitrogen-Thermo Fisher Scientific, Waltham, MA, USA). Membranes were scanned at 700 nm and 800 nm, respectively, using the Odyssey infrared imaging system (LI-COR Biosciences, Lincoln, NE, USA). Images were treated and proteins were quantified with the Odyssey software. The amounts of phospho-RAD51 on the blot were normalized to the amount of RAD51 and the ratio of phospho-RAD51 to total RAD51 was determined from three independent experiments.

### 4.9. Blitz® DNA Binding Assay

Biotinylated DNA polydT (100 mM) was bound on a streptavidin coated biosensor (tip). RAD51 proteins (4 µM) were prepared in reaction buffer made of PBS buffer 1× and ATP 2 mM. Kinetics were divided in three steps. Firstly, baseline of the buffer was measured for 10 s, then the association step between ssDNA and RAD51 at 4 µM was monitored for 200 s and finally the dissociation step of the bound RAD51 was monitored for 200 s in buffer with or without BRC4. Tip was regenerated in NaOH (50 mM) twice for 40 s before reuse.

### 4.10. D-Loop Assay

100-ssDNA labeled IRD700 (1 μM) (Integrated DNA Technologies, Coralville, IA, USA) was incubated with 0.5 μM RAD51 in the presence or absence of the indicated amounts of the corresponding molecule in 10 μL of standard reaction buffer containing 20 mM Tris–HCl (pH 8), 1 mM ATP, 1 mM DTT, 1 mM CaCl_2_ at 37 °C for 20 min. The reaction was initiated by adding supercoiled pPB4.3 DNA (200 μM in bp). After incubation of 30 min at 37 °C, the reactions were stopped and deproteinized by a stop solution (10 mM Tris–HCl pH 8, 10 mM MgCl_2_, 1% SDS, and 1 mg/mL proteinase K). The reaction mixtures were further incubated for 15 min at 37 °C. After adding 5-fold loading dye (0.05% bromophenol blue, 8% glycerol, 1 mM EDTA), the reaction products were separated by electrophoresis on 1% agarose gel. The electrophoresis was carried out in 0.5× TAE buffer (20 mM Tris, 10 mM acetic acid and 1 mM EDTA) at 100 V for 2 h. The labeled products (100-ssDNA and D-loop) were visualized and quantified by the detection of the IRD-700 dye with the 700 nm infrared fluorescent detection channel of an Odyssey Infrared Imager (LI-COR Biosciences) [47].

### 4.11. HR-GFP Assay

RG37 cells stably expressing the direct-repeat (DR)-GFP construct were generously given by Dr LOPEZ B. (IGR, Paris, France). RG37 cells were plated into 12-well plates, 1 day later pretreated by HGF (50 ng/mL) and subsequently transiently transfected with pCBA-HA-I-SceI (functional endonuclease) using Lipofectamine2000 (#11668019, Invitrogen). Transient expression of I-SceI endonuclease generated a DNA double strand break at the integrated GFP gene sequences and stimulated HR. GFP signal was assayed 72-hr post-transfection on a CytoFLEX flow cytometer (Beckman Coulter, Brea, CA, USA). For each experiment, 10,000 cells were scored per treatment group, and the recombination percentage was calculated in comparison with the number of GFP-positive cells of the control condition RG37 I-SceI.

### 4.12. Statistical Analysis

Statistical analysis was performed using paired Student’s *t*-test. Statistical significance was assumed at * *p* < 0.05, ** *p* < 0.01, *** *p* < 0.001 or Non-Significant NS for *p* > 0.05. Each error bars represents s.d. Statistical analysis and signal values representations were performed using the Microsoft Excel 2013 software.

## 5. Conclusions

This study demonstrates the direct link between RAD51 and c-MET wherein the phosphorylation of RAD51 impact its oligomeric state but also its interaction with the BRC motif of BRCA2. Our results suggest that c-MET-mediated RAD51 phosphorylation could play a potential role in the BRCA2-dependent regulation of RAD51. Given our encouraging in vitro findings, the next step should be to expand our investigations in cells to assess the RAD51 phosphorylation level according to c-MET inhibition in the DNA damage response pathway. The expected results could pave an attractive therapeutic strategy to overcome resistance by combining the modulation of c-MET and RAD51 activity with anticancer treatments.

## Supplementary Materials

The following are available online at https://www.mdpi.com/2072-6694/11/3/413/s1, Figure S1: Purification of RAD51 mutants, Figure S2: CD spectra of ten RAD51 Y/F mutants and RAD51-wt.

## Abbreviations

AKT/PKB	protein kinase B
BRCA2	Breast Cancer 2
BRC4/BRC4-28	28 aa of the BRC motifs
c-MET: HGFR	hepatocyte growth factor receptor
DSB	double strand break
EGFR	Epidermal growth factor receptor
GAB1	GRB2 Associated Binding Protein 1/Growth Factor Receptor Bound Protein 2-Associated Protein 1
HGF	Hepatocyte Growth Factor
HR	Homologous recombination
MAPK	Mitogen-activated protein kinases
PI3K	phosphoinositide 3-kinase
pRAD51	phospho-RAD51
RTK	Receptor tyrosine kinase
STAT3	Signal transducer and activator of transcription 3
ssDNA	single strand DNA
