# Supplementary material for: New Phosphorylation Sites of Rad51 by c-Met Modulates Presynaptic Filament Stability
Source: Cancers (Basel). 2019 Mar 23;11(3):413. doi: 10.3390/cancers11030413 (PMC6468871; doi:10.3390/cancers11030413)
Supplement: Supplementary file 1 [file cancers-11-00413-s001.pdf]

Supplementary Materials: New Phosphorylation Sites of Rad51 by c-Met Modulates Presynaptic Filament Stability

Thomas Chabot, Alain Defontaine, Damien Marquis, Axelle Renodon-Corniere, Emmanuelle Courtois, Fabrice Fleury and Yvonnick Cheraud

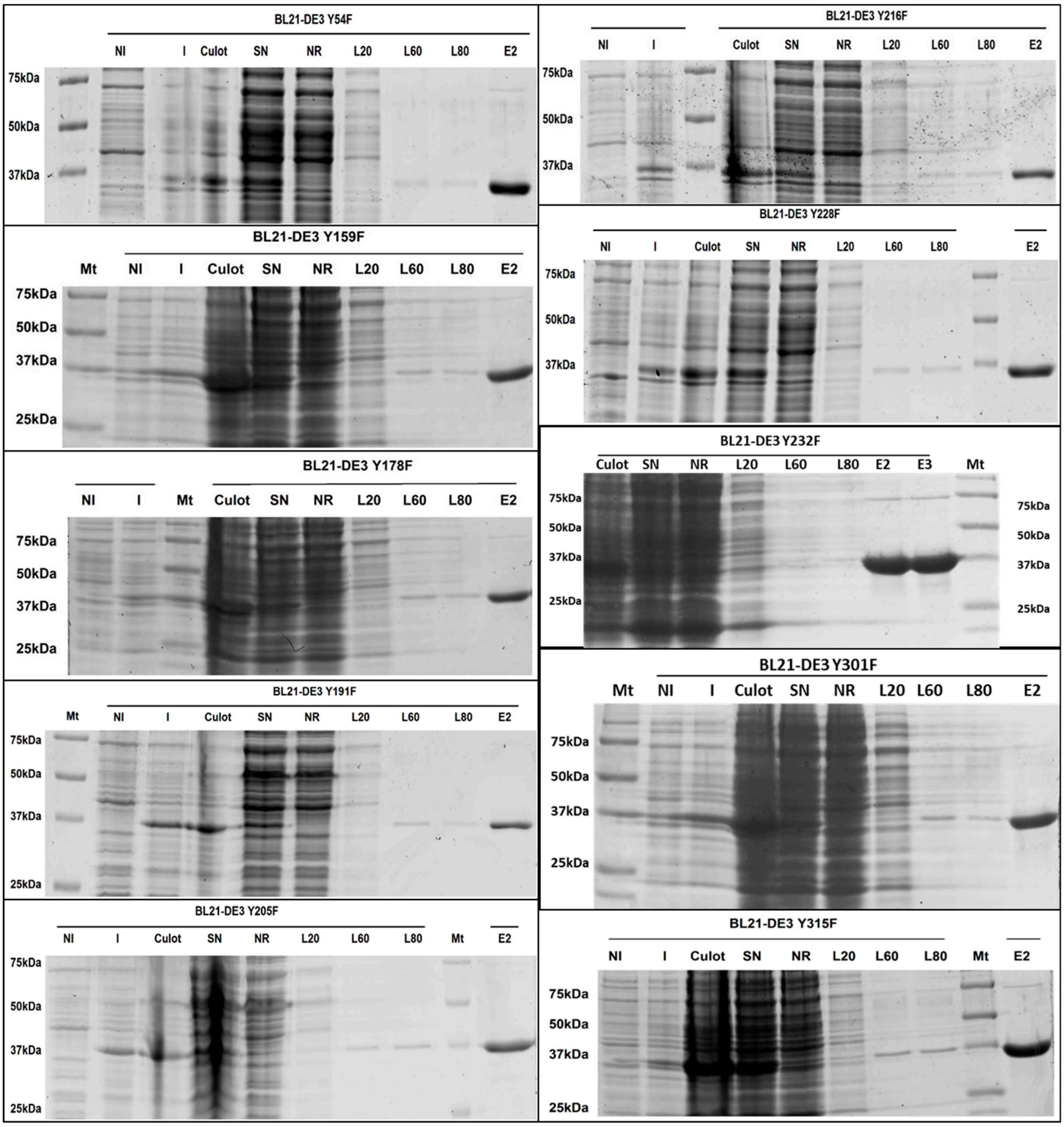

Figure S1. Purification of RAD51 mutants. The DNA sequence contructs of the mutant clones were checked and validated before and after induction with IPTG (lanes NI and I). RAD51-WT and RAD51 mutants were then over-expressed in Escherichia coli BL21- DE3 strain at 37°C. The bacteria are then lysed and His-tagged RAD51-proteins were purified on a NiNTA resin. Each blot corresponds to a mutant His-RAD51 protein expressed in BL21-DE3. On each of the gels are represented the extracts obtained after each of the protein purification steps. (NI: Non-Induction ; I: Induction Protein Synthesis ; SN: Bacterial Lysate ; NR: Bacterial Fraction not retained by resin ; L20/60/80: Washing with increasing concentration Imidazole in mM ; E: Elution).

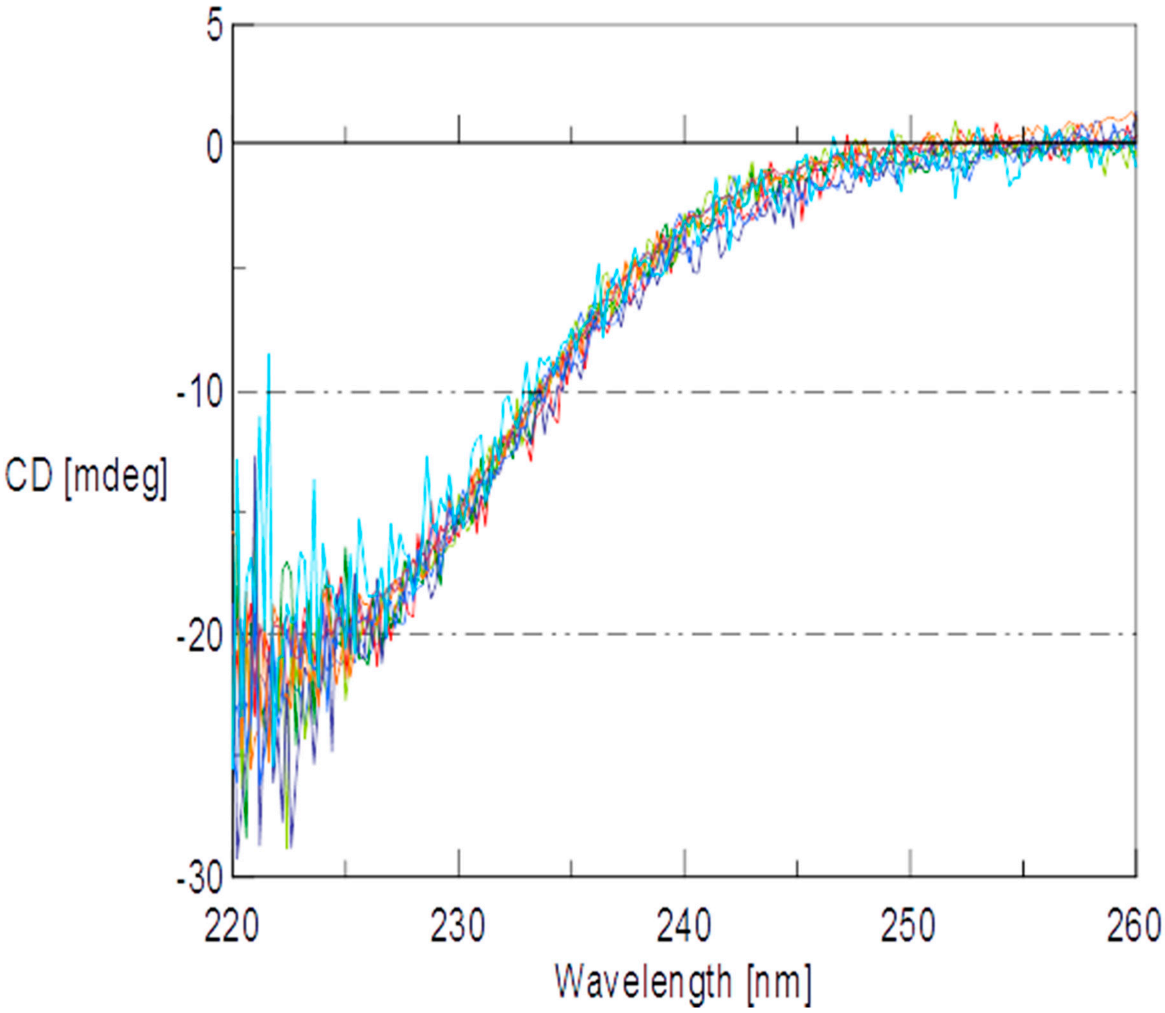

Figure S2. CD spectra of ten RAD51 Y/F mutants and RAD51-wt. The signals were obtained at a constant temperature of 20°C and were measured from 220 to 260 nm in a Hellma quartz cell with an optical distance of 0.2 cm. The final concentration of each RAD51 protein was 4 μM in phosphate buffer saline (PBS), pH 7.4. The ellipcity at 222 nm correspond to helix content of protein. The CD signals at 222 nm are almost identical and the profile comparison of the ten CD spectra shows that the secondary structure of recombinant Rad51 mutants is similar to the Rad51-wt.
